# Double-Layer Agar (DLA) Modifications for the First Step of the Phage-Antibiotic Synergy (PAS) Identification

**DOI:** 10.3390/antibiotics10111306

**Published:** 2021-10-26

**Authors:** Xymena Stachurska, Marta Roszak, Joanna Jabłońska, Małgorzata Mizielińska, Paweł Nawrotek

**Affiliations:** 1 Nanotechnology Center for Education and Research, Department of Microbiology and Biotechnology, Faculty of Biotechnology and Animal Husbandry, West Pomeranian University of Technology in Szczecin, Piastów Avenue 45, 70-311 Szczecin, Poland; pawel.nawrotek@zut.edu.pl; 2 Department of Laboratory Medicine, Chair of Microbiology, Immunology, and Laboratory Medicine, Pomeranian Medical University in Szczecin, Powstańców Wielkopolskich Avenue 72, 70-111 Szczecin, Poland; marta.roszak@pum.edu.pl; 3 Department of Chemical Engineering, Faculty of Chemical Technology and Engineering, West Pomeranian University of Technology in Szczecin, Piastów Avenue 42, 71-065 Szczecin, Poland; joanna_jablonska@zut.edu.pl; 4 Center of Bioimmobilisation and Innovative Packaging Materials, Faculty of Food Sciences and Fisheries, West Pomeranian University of Technology in Szczecin, Janickiego 35, 71-270 Szczecin, Poland; malgorzata.mizielinska@zut.edu.pl

**Keywords:** antibiotics, bacteriophage, phage-antibiotic synergy, *Escherichia coli*, double-layer agar method

## Abstract

The research carried out so far for phage-antibiotic synergy (PAS) differs as regards the technique of modifying the double-layer agar (DLA) method to show the PAS effect on Petri plates, which may contribute to non-uniform research results. Therefore, there is a need to unify the method to effectively detect the PAS effect, at its most basic in vitro test. In this study, bacteriophage T4_5_ and 43 antibiotics belonging to different antibiotic classes were used. Seven different DLA method modifications were tested, in terms of antibiotic addition placement and presence or absence of the base agar. The overall number of phage plaques per plate mainly depended on the antibiotic used. Differences in plaque quantity depended on the type of the DLA method modification. The largest total number of plaques was obtained by the addition of an antibiotic to a bottom agar with the presence of a top agar. This indicates that even though an antibiotic could manifest the PAS effect by a standard disk method, it would be worth examining if the effect is equally satisfactory when applying antibiotics directly into the agar, with regards to using the same bacteriophage and bacterial host.

## 1. Introduction

With the increase in the frequency of antibiotic resistance and the decreasing numbers of new antibiotics being developed [1], alternative strategies for antibiotic therapy are urgently needed by the worldwide medical and scientific community. Bacteriophages have recently gained interest as therapeutic agents [2].

Phage therapy is based on the use of lytic phages to combat bacterial infections, including multidrug-resistant bacteria, and has many advantages, as phages persist as long as the targeted bacteria are present. Moreover, phages are very specific and efficient for their bacterial host. This reduces the destruction of natural flora and presents no detrimental effects on human cells [3]. Therefore, some researchers have become interested in using phages in combination with modern antibiotics and have demonstrated that an approach of using phages, along with the antibiotics can be a promising way to improve antimicrobial activity [4,5,6,7,8,9].

This phenomenon is known as phage-antibiotic synergy (PAS), given this name by the authors who first described this effect [10]. The PAS effect occurs when sub-lethal concentrations of certain antibiotics substantially stimulate a host bacterium to produce virulent phage [4,10]. Phages may offer the much-needed potential to complement antibiotics, mostly due to differences in their modes of action but also because of the countless diversity of the phages. The logic of combining phages and antibiotics stems from an evolutionary understanding that two sufficiently different selective pressures are likely to be more effective than either alone [11].

The expected benefit of such a dual approach might be enhanced bacterial suppression, more efficient penetration into biofilms, and a decrease in the emergence of resistance [12]. The last point seems to be the most interesting, since it has been demonstrated that phage selection pressure on multi-drug-resistant (MDR) *P. aeruginosa* produces an evolutionary trade-off, whereby the evolution of bacterial resistance to phage attack changes the efflux pump mechanism, causing increased sensitivity to drugs from several antibiotic classes [13]. Hypothetically, this promising result might also occur in other bacterial pathogens with similar modes of achieving broad antibiotic resistance [14].

Although the lytic activity of phages has been shown to be synergistically enhanced in the presence of antibiotics, PAS is still not fully understood. The research carried out to date differs as regards the method of modifying the double-layer agar (DLA) method to demonstrate the PAS effect [15,16], which may contribute to non-uniform research results at the most important method level. Moreover, DLA phage assay methodology can direct the choice of phage-antibiotic combinations for further testing of this phenomenon. Therefore, there is a need to unify a method of effectively detecting the PAS effect in a first, most basic in vitro test on Petri plates.

The purpose of the study was to standardize the double-layer agar (DLA) method on Petri plates for T4-like phages, in order to evaluate the most efficient variable for phage-antibiotic synergy (PAS) effect detection.

## 2. Results

### 2.1. Antibiotic Selection to Enable the Occurrence of the PAS Effect

**Figure 1:**
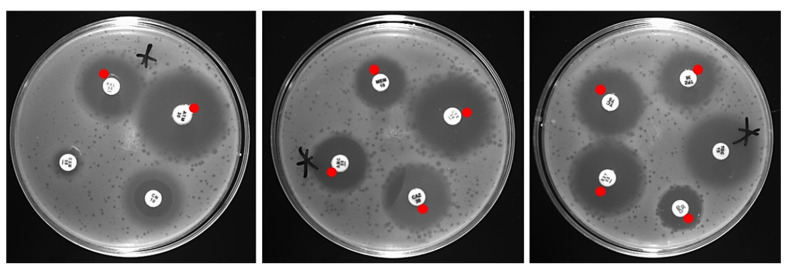
Sample visualization of the occurrence of the PAS effect. Antibiotics classified as demonstrating the potential for the PAS effect (marked with red dots) were characterized as showing larger phage plaques in zones surrounding antibiotic disks where there was a sub-lethal concentration of the drug, compared to the standard T4_5_ plaques. A star asterisk indicates plates where the PAS effect was present.

The experiments were performed relying on guidelines and based on the findings of Comeau et al. [10]. The results of the study determined: the high possibility of the occurrence of the PAS effect (++), by detecting an increased amount of morphologically larger (than standard) T4_5_ plaques, in the bacterial inhibition zones caused by the antibiotic; the possibility of the occurrence of the PAS effect (+), by detecting the appearance of the larger plaques in the sub-lethal zones; (edge of the zones surrounding antibiotic disks where there is a sub-lethal concentration of the drug) no PAS effect (/), when plaques had a standard size of T4_5_ bacteriophage and plaque quantity had not changed; possible antagonist effect (-), when there were less morphologically standard plaques within the sub-lethal drug zones (Figure 1).

The results of this experiment are shown in Table 1. From 43 antibiotics used for PAS effect determination, two β-lactams (cefotaxime and ceftazidime) demonstrated a high possibility that the PAS effect would occur. Also, ampicillin was characterized as provoking PAS induction (data not shown). A total of 14 antibiotics demonstrated the possibility of the occurrence of the PAS effect by the appearance of some larger plaques in the sub-lethal zones. They were as follows: (β-lactams) aztreonam, amoxicillin/clavulanic acid, piperacillin, cefoperazone, piperacillin/tazobactam, ticarcillin, meropenem, cefoxitin, (fluoroquinolones) marbofloxacin, flumequine, ciprofloxacin, (polymyxin antibiotic) colistin sulfate, and (sulfonamide with DHFR inhibitor) trimethoprim-sulfamethoxazole. A possible antagonistic effect was detected with 6 antibiotics: (tetracyclines) tigecycline, tetracycline, doxycycline, (amino-glycosides) streptomycin, kanamycin, and gentamicin. Other antibiotics showed no effect (Table 1).

Cefotaxime and ampicillin were selected for further experimentation.

### 2.2. Antibiotic Susceptibility Assay

The antibiotic susceptibilities of *E. coli* were determined according to Wiegand et al. [17]. MICs of cefotaxime and ampicillin were tested in ranges of 25,000–0.00017 µg/mL and 25,000–48.83 µg/mL, respectively. The mean of the MICs of cefotaxime, observed for the bacterial strain, was 0.0225 µg/mL and the mean of the MICs of ampicillin was 390.625 µg/mL. Since the synergy is observed as significantly larger phage plaques in zones surrounding antibiotic disks where there is a sub-lethal concentration of the drug, the optimal antibiotic concentrations for effective PAS were deduced to be sub-MIC [4]. These concentrations were set up to 0.5 × MIC—for cefotaxime 0.011 µg/mL and for ampicillin 195.3 µg/mL.

### 2.3. Double-Layer Agar (DLA) Method Variables

Variables of the DLA method contained different variations of layers and different types of antibiotic additions in order to examine how it would affect plaque formation and PAS effect generation by the T4_5_ bacteriophage. Five different method models were tested in this study with suitable controls, giving seven different variables. Antibiotics were added in their sub-MIC concentrations, being 0.011 µg/mL for cefotaxime and 195.3 µg/mL for ampicillin, or in the form of antibiotic disks. Photographs visualizing the results on Petri dishes—showing plaque sizes with their arrangement, were added to the Supplementary Materials (Figures S1 and S2).

**Figure 2:**
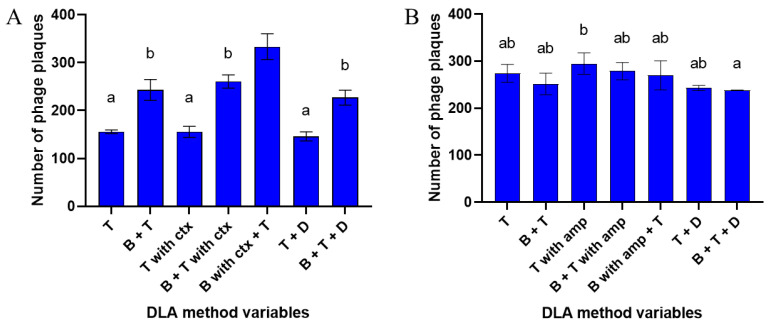
The total number of phage plaques per plate depending on the DLA method variable with the addition of (**A**) cefotaxime or (**B**) ampicillin. Top agar was marked as “T”, bottom agar as “B”, antibiotic disk as “D”, cefotaxime and ampicillin as “ctx” and “amp”, respectively. Phage was added to the top agar layers. Vertical bars indicate means ± SD. a, b—means sharing the same superscript are not significantly different from each other at *p* ≤ 0.05.

The overall number of phage plaques per plate depended mainly on the antibiotic used. The ampicillin test (Figure 2B) showed less differentiation between results from different method modifications, as opposed to the cefotaxime test (Figure 2A). The addition of ampicillin showed no significant differences in plaque enumeration in any of the method modifications, compared to the control with no antibiotic, despite being able to trigger the PAS effect (Figure S1). However, the addition of cefotaxime caused significant changes in the number of plaques, depending on the type of DLA method modification. The largest total number of plaques was obtained during the addition of the antibiotic to the bottom agar with the presence of the top agar (“B with ctx + T”) (Figure 2A). This modification increased the number of plaques by 114% compared to the use of top agar alone (“T”) and by 37% compared to the standard DLA method containing bottom and top agar (“B + T”).

**Figure 3:**
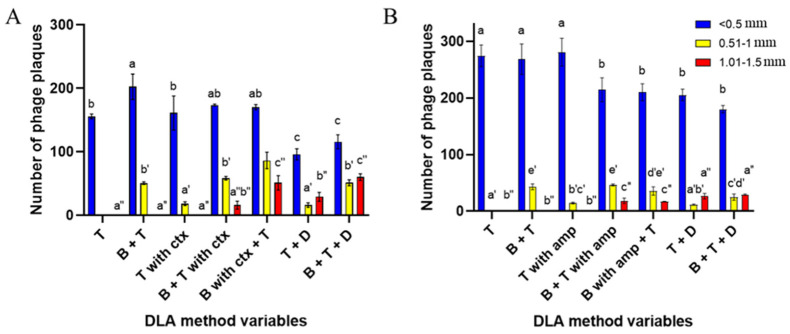
The number of phage plaques per plate divided into diameter ranges, depending on the DLA method variable with the addition of (**A**) cefotaxime or (**B**) ampicillin. Diameter ranges were established as follows: <0.5 mm—small, standard T4 plaques (blue bars), 0.51–1 mm—medium, standard T4 plaques (yellow bars), 1.01–1.5 mm—large, strong PAS-indicating plaques (red bars). Top agar was marked as “T”, bottom agar as “B”, antibiotic disk as “D”, cefotaxime and ampicillin as “ctx” and “amp”, respectively. Vertical bars indicate means ± SD. a, b, c, …, a’, b’, c’, …, and a’’, b’’, c’’, …,—means sharing the same superscript are not significantly different from each other at *p* ≤ 0.05.

The results in the number of phage plaques divided into diameter ranges showed statistically significant differences between the type of method used and plaque sizes (Figure 3A,B). Overall, the addition of cefotaxime resulted in a larger amount of standard T4_5_ plaques of medium size (0.51–1 mm) and large, strong PAS-indicating plaques (1.01–1.5 mm) than in the presence of ampicillin but smaller amounts of small, standard T4_5_ plaques (<0.5 mm) compared to AMP addition. Furthermore, the best results were also obtained during the addition of the antibiotic to the bottom agar with the presence of the top agar (“B with ctx + T”) and with the application of the antibiotic disk onto the standard soft-agar overlay with the bottom layer present (“B + T + D”). For ampicillin, “B + T with amp” and “B with amp + T” modifications were equally effective in terms of forming different diameter plaques. For big, PAS-indicating plaque (0.51–1 mm) visualizations, antibiotic disk modifications (“T + D” and “B + T + D”) showed equally best results. Only the “B + T + D” variable was characterized by a greater amount of medium-sized, standard phage plaques due to the presence of a bottom layer (Figure 3B). Nevertheless, the addition of the liquid antibiotic, regardless of the modification method used, increased the overall number of medium-sized plaques, where the application of the antibiotic disks increased the overall number of large, PAS-indicating plaques.

## 3. Discussion

Phage-antibiotic synergy was discovered many years ago, however, this effect was not fully characterized at that time and did not have its own name [18,19,20]. In the cited studies, antibiotics have been found to simply influence phage growth, for example, more phage formed in the presence of penicillin, or beta-lactam antibiotics stimulating phage development in *Escherichia coli* and *Staphylococcus aureus*. In other more recent research, antibiotics were used to improve phage detection and enumeration, or for plating, phages forming very small plaques or no plaques under standard conditions by adding different antibiotics to the agar media in order to obtain plaques of larger diameter [15,16]. In the meantime, the PAS effect has been defined and described [10], which initiated interest in studies on the possible co-use of antibiotics and phages, primarily in terms of observing their synergies and additive effects. However, studies on the synergy effects with the use of the DLA technique differ in the way antibiotics are implemented [15,16]. That may have contributed to the non-uniform research results at the most important, method level. Taking this into consideration, an attempt was made to unify and standardize the method of effectively detecting the PAS effect at the initial step for T4-like bacteriophages; the most basic in vitro test on Petri plates.

Within this research, 43 antibiotics were used. The antibiotics belong to different classes (Table 2) because antibiotics with similar modes of action may result in different outcomes when combined with specific phages [21]. This statement is in line with the present study, where it was noted that three antibiotics from the β-lactam class (cefotaxime, ceftazidime, and ampicillin) demonstrated a very high possibility of synergy and 14 other antibiotics from different classes showed a likelihood of the existence of PAS—due to the probability of the appearance of the additive effect (Table 1). These were as follows: (β-lactams) aztreonam, amoxicillin/clavulanic acid, piperacillin, cefoperazone, piperacillin/tazobactam, ticarcillin, meropenem, cefoxitin, (fluoroquinolones) marbofloxacin, flumequine, ciprofloxacin, (polymyxin antibiotic) colistin sulfate (sulfonamide with DHFR inhibitor), and trimethoprim-sulfamethoxazole.

Comeau et al. [10] also reported the presence of the PAS effect on an *E.coli* strain with the addition of disks of cefotaxime and ceftazidime but with the use of *Siphoviridae* phage. In the experiments of others, there was also an increase in the T4-like phage plaques diameters in the presence of ampicillin, which was also found in the present study but also in the presence of kanamycin, tetracycline, and chloramphenicol [16]. The results of this study demonstrated that the application of chloramphenicol did not present any particular effect, while kanamycin and tetracycline showed a possible antagonistic outcome. These differences may be caused by a different approach in antibiotics incorporation. The experiments were carried out using a standard DLA method with the addition of antibiotics in the form of disks placed on top of the soft-agar as described by Comeau et al. [10]. On the other hand, in research conducted by Łoś et al. [16] antibiotics were applied directly into the base-agar. Ciprofloxacin, which showed the probable appearance of additive effects in the experiment, also displayed a synergistic effect in *E. coli* inactivation combined with T4-like phage in the liquid culture [22]. The PAS effect was also reported to enhance biofilm removal by reducing the minimum biofilm eradication concentration value of cefotaxime against *E. coli* biofilms from 256 to 128 and 32 µg/mL when the medium (10^4^ PFU/mL) and high (10^7^ PFU/mL) T4 phage titers were added [4].

Due to the results of other authors [4,10] with cefotaxime successively recurring in many studies focusing on PAS, it was selected for further experiments. As cefotaxime was often tested and its PAS evocation is confirmed, it was found to be the best choice for the evaluation of the phage-antibiotic synergy effect with different variables of the double-layer agar method. Ampicillin was utilized as it showed the induction of the PAS effect similarly to cefotaxime, which was also confirmed in the results of other authors [16]. Ampicillin is also a widely used drug to treat *E. coli* infections [23]. Antibiotics susceptibility assay was conducted in order to acquire MICs, which were necessary to determine 0.5 × MIC (sub-MIC) concentrations of antibiotics since synergy was originally observed as significantly larger phage plaques in zones surrounding antibiotic disks, where there is a sub-lethal concentration of the drug. 0.5 × MIC was 0.011 µg/mL for cefotaxime and 195.3 µg/mL for ampicillin.

**Figure 4:**
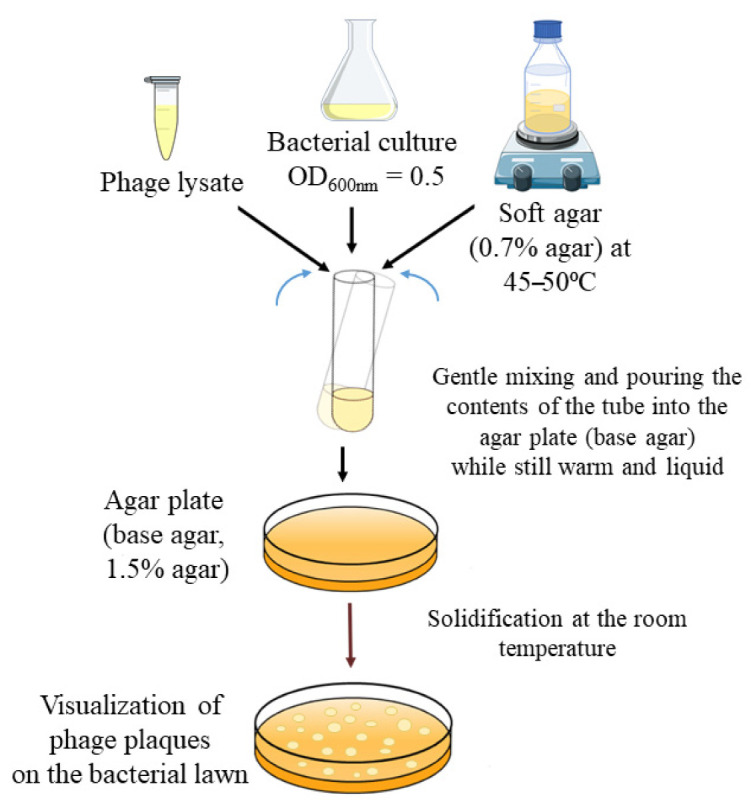
Standard double-layer agar (DLA) method visualization (prepared based on the methodology described by Kropinski et al. [24]).

The final experiment consisted of testing different DLA method variables in order to standardize and evaluate which was the most efficient for phage-antibiotic synergy (PAS) effect detection. Standard “double-layer agar method” or “double overlay agar plaque assay” is used for the determination of the concentration of functional bacteriophage particles (titre), usually expressed as plaque-forming units (PFU/mL). The method is based on mixing phage suspension (lysate) dilutions with host bacteria in “soft” agar, distributing this mixture on a standard agar plate to solidify. After incubation (with conditions adapted to the host bacterium), plaques are visualized as clear zones in the bacterial lawn (Figure 4). The DLA method is used as the basis for the isolation of phages and their characterization (clear or turbid plaques, plaque sizes, and the presence/absence of a halo) [24].

Within the research, seven different DLA method modifications were tested in terms of antibiotic addition placement, as well as the presence or absence of the base agar with proper control plates. The addition of the antibiotic as antibiotic disks applied onto Petri plates was carried out as this was the first method used for the original PAS detection [10] and was successfully repeated by other authors [25,26]. The addition of an antibiotic to the soft LB agar was conducted because this was shown to be effective in many other studies [4,7,10,27], and the incorporation of the antibiotic to the base agar was added to allow a slow diffusion of the drug from the bottom layer into the bacterial growth zone [16]. Variables without the base agar were included because in some circumstances, the lower layer in the DLA method is skipped, and the semi-solid media may be poured directly into the plate, though this approach requires thicker agar layers [28].

The results of the study showed that the overall number of phage plaques per plate depended mainly on the antibiotic used. Despite triggering the PAS effect, the addition of ampicillin showed no significant differences in plaque enumeration in any of the method modifications that were used. However, in the case of cefotaxime, significant changes in the overall number of plaques were detected. Differences in the number of plaques depended on the type of the DLA method modification. The largest total number of plaques was obtained by the addition of an antibiotic to the bottom agar with the presence of the top agar, where bacterial and phage suspensions were mixed. This modification increased the number of plaques by 114% compared to the use of a top agar alone and by 37% compared to the standard DLA method containing bottom and top agar. In the case of the number of phage plaques divided into diameter ranges, experiments showed statistically significant differences between the type of method used and the antibiotic on plaque sizes. Cefotaxime incorporation resulted in a greater amount of large, strong PAS-indicating plaques than in the presence of ampicillin, with the best results also obtained with the addition of the antibiotic to the bottom agar with the presence of the top agar, beside the antibiotic disk modification with the bottom layer. For ampicillin, big PAS-indicating plaques were most visible with antibiotic disk modifications, regardless of the presence of a bottom layer.

The results of this study indicate, that even though an antibiotic can manifest the PAS effect with T4-like phage by the standard disk method, further examination should be carried out to verify if the effect will be equally satisfactory when applying the antibiotic directly into the agar. One of the antibiotics used enhanced the total number of phage plaques (cefotaxime), where the other did not (ampicillin), despite belonging to the same antibiotic class (β-lactams). However, ampicillin is a member of the penicillins group, and cefotaxime belongs to the cephalosporins, and as reported by other authors, phage and antibiotic synergistic and antagonistic interactions depend highly on the mechanism of bacterial inhibition and stoichiometry of the pairing [29], and cefotaxime tends to have a broader spectrum of activity compared with ampicillin [30]. Also, the choice of the DLA method variable seems to be mostly antibiotic type-dependent with regards to using the same bacteriophage and bacterial host. In order to visualize the PAS effect, disk modification showed the best results, where there was an overall increase in the different diameter phage plaques due to the application of the antibiotic directly into the bottom agar layer, which could be primarily due to the slow diffusion of the drug from the bottom layer into the zone of bacterial growth.

## 4. Materials and Methods

### 4.1. Bacterial Strains and Bacteriophage Propagation

*Escherichia coli* K-12 C600 [31] was used as a host strain for the propagation of bacteriophage T4_5_ (own isolate, T4-like phages representative). Phage and bacterium were part of a Collection of the Department of Microbiology and Biotechnology, at the West Pomeranian University of Technology, Szczecin. The bacterium was stored using the Microbank™ system at −20 °C prior to the experiments. In order to recover the strain, beads were streaked directly to Luria-Bertani (LB agar) (BioMaxima, Lublin, Poland) plates and incubated at 37 °C for 24 h. Next, a single colony was streaked onto a new LB agar plate with the same incubation conditions and then used to inoculate 50 mL of LB broth. Bacterial cells were incubated at 37 °C and 160 rpm in an orbital rotating shaker (Shaker-Incubator ES-20, BioSan, Józefów, Poland) to reach OD_600nm_ = 0.5, corresponding to the exponential phase of growth and a titer of ~2 × 10^8^ CFU. Optical density values were measured using an Infinite 200 PRO NanoQuant microplate reader (Tecan, Männedorf, Switzerland). The *E. coli* bacteriophage T4_5_ was added at this point, and samples were incubated for the further stage until the cultures cleared, or after 3 h. Following incubation, chloroform was added to the culture (10%, *v*/*v*), and samples were vortexed for the next 5 min. The cultures were then centrifuged (Eppendorf Centrifuge model 5810 R, Hamburg, Germany) at 3500× *g* for 15 min at 4 °C, and the supernatant was collected and stored at 4 °C. Phage activity was tested by a double overlay agar plaque assay [24]. The clear plaque was picked with a pipette tip, and phages were suspended in TM buffer (50 mM Tris-HCl, 10 mM MgSO_4_ at pH 7.5). Isolated phages were purified through a triple transfer of single plaque and propagated until homologous plaques were obtained. Decimal dilutions of lysates in a TM buffer were prepared to define the titer of the bacteriophage and subjected to the plaque assay using the double agar layer method. Plaques were counted on a dilution plate in spots containing 30–300 plaques. Results were expressed in plaque-forming units per mL. Bacteriophage stock suspension was stored at 4 °C.

### 4.2. Antibiotics Used for PAS Effect Occurrence

In order to detect which antibiotics may form the PAS effect, 43 different antibiotics were used (Table 2). The test was performed using a double-layer agar method combined with an antibiotic susceptibility assay with the use of a disk diffusion test in accordance with Clinical and Laboratory Standards Institute (CLSI) recommendations. Base LB agar (1.5%) plates were covered with top LB agar (0.7%) containing 500 µL of bacterial culture (OD_600nm_ = 0.5) and 100 µL of phage lysate (of approx. 1 × 10^7^ PFU/mL). After top agar solidification, the antibiotic disks were applied. Plates were incubated for 24 h at 37 °C and then analyzed for the presence of the PAS effect. The presence of the PAS effect was determined according to Comeau et al. [10]. Synergy was observed as significantly larger phage plaques compared to the control, in zones surrounding antibiotic disks where there was a sub-lethal concentration of the drug [10].

### 4.3. Antibiotics Susceptibility Assay

Based on the previous experiment, cefotaxime and ampicillin were chosen for further testing. Ampicillin and cefotaxime (A&A Biotechnology, Gdańsk, Poland) stock solutions of 50 mg/mL were prepared by diluting them in deionized water and filter sterilizing (0.2 µm; PES 25 mm syringe filter; VWR International, Radnor, PA, USA). The MICs of antibiotics were determined by using a 96-well microplate dilution protocol with minor modifications [17]. LB was used as the main medium in the test and freshly inoculated bacterial culture was incubated till it reached OD_600nm_ = 0.2 (which corresponds to 1 × 10^8^ CFU/mL). The bacterial suspension was then diluted at 1:100 in the LB medium, which gave the final desired inoculum of 5 × 10^5^ CFU/mL. The microtiter plate was incubated at 37 °C for 24 h. The MIC of the antibiotics for *E. coli* K-12 C600 bacterial strain was determined to be a concentration of antibiotic at which the optical density (OD_600nm_) was equal to that of a cell-free blank control.

### 4.4. Double-Layer Agar (DLA) Method Variables

**Figure 5:**
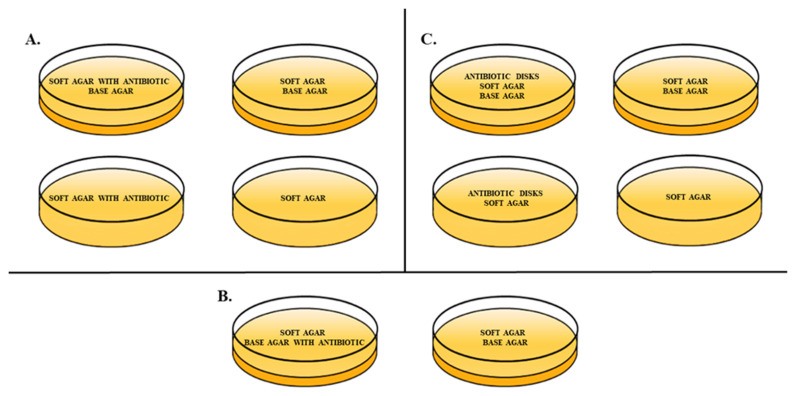
Types of DLA method modifications used in terms of antibiotic addition placement and the presence of a base agar with proper control plates; (**A**) addition of the antibiotic to the soft LB agar with and without base agar with control plates of a standard DLA method and soft agar, (**B**) addition of the antibiotic to the base LB agar with a standard DLA method control plate, (**C**) addition of the antibiotic as antibiotic disks applied onto plates with soft and base LB agar, or with only the soft LB agar with control plates of a standard DLA method and soft agar.

For the experiment, different variables of the double-layer agar method [24] were used in order to assess the presence of the phage-antibiotic synergy effect (Figure 5A–C). Various types of DLA methods included: adding the antibiotic to the soft agar, with and without base agar; adding the antibiotic to the base agar; and finally applying antibiotic disks onto plates with soft and base agar, or with only soft agar. Antibiotics in a liquid form were added to the agar media in concentrations of 0.5 × MIC. Proper control plates of the standard DLA method and soft agar were also conducted. Additionally, 5 mL of soft agar containing 100 µL of phage lysate was added at a titer of approx. 9.3 × 10^3^ PFU/mL and 200 µL of *E. coli* was added when bacterial cells were in their exponential (log phase) of growth—at OD_600nm_ = 0.5. The experiment was carried out in triplicate.

### 4.5. Statistical Analysis

The results were analyzed statistically with Statistica 13.3 TIBCO Software Inc. (StatSoft Inc., Tulsa, OK, USA). Shapiro–Wilk tests were used to evaluate the distribution of variables. One-way ANOVA was performed, and the significance of differences between the mean values of the number of phage plaques and the variables of the DLA method were calculated using a parametric Tukey test. Differences were considered significant at *p* ≤ 0.05.

## 5. Conclusions

In this work we tried to unify the DLA method to effectively detect the PAS effect for T4-like bacteriophages, at its most basic in vitro test. Our studies have shown that the choice of the method modification has an impact on the final results. The selection of the DLA method variable seems to be mostly antibiotic type-dependent with regards to using the same bacteriophage and bacterial host. This indicates that even though an antibiotic could manifest the PAS effect by a standard disk method, it would be worth examining if the effect is equally satisfactory when applying antibiotics directly into the agar.

## Figures and Tables

**Table 1:** Antibiotics used in the determination of the occurrence of PAS effect: (++) high possibility of PAS effect occurrence, (+) possibility of PAS effect occurrence, (/) no effect, (-) possible antagonist effect.

Antibiotic (Abbreviation and Concentration)	Probability of the PAS Effect Occurrence	Antibiotic (Abbreviation and Concentration)	Probability of the PAS Effect Occurrence	Antibiotic (Abbreviation and Concentration)	Probability of the PAS Effect Occurrence
ATM 30	+	AMC 30	+	UB 30	+
PRL 30	+	CTX 30	++	DA 2	/
CN 10	-	CAZ 30	++	VA 30	/
CXM 30	/	CPD 30	+	CFP 75	+
NOR 10	/	SXT 25	+	CL 30	/
AML 25	/	TGC 15	-	TE 30	-
TOB 10	/	TPZ 36	+	LNZ 30	/
AK 30	/	TC 75	+	FA 10	/
SYN 15	/	MEM 10	+	RA 5	/
AUG 3	/	FOR CYL	+	TEC 30	/
CEF 30	/	DO 30	-	S 300	-
OB 5	/	OX 1	/	K 30	-
C 30	/	E 15	/	CIP 5	+
FOX 30	+	P 10	/	MY 15	/
CT 25	+	OT 30	/		

**Table 2:** Antibiotics used in the PAS effect presence determination.

Mode of Action	Antibiotic Class	Antibiotic	Antibiotic Abbreviation	Company of Origin
Inhibition/disruption of the cell wall synthesis	*β*-lactams (penicillins, cephalosporins, cephamycins, carbapenems, monobactams)	Piperacillin	PRL	Oxoid
		Amoxicillin	AML	Oxoid
		Amoxicillin/clavulanic acid	AMC	Oxoid
		Penicillin G	P	Oxoid
		Cloxacillin	OB	Oxoid
		Oxacillin	OX	BioMaxima
		Ticarcillin	TC	Oxoid
		Piperacillin/tazobactam	TPZ	Oxoid
		Cefacetril 30 masticef	CEF	BioMaxima
		Ceftazidime	CAZ	BioMaxima
		Cephalexin	CL	Emapol
		Cefoperazone	CFP	Oxoid
		Cefuroxime	CXM	Oxoid
		Cefotaxime	CTX	BioMaxima
		Cefpodoxime	CPD	Emapol
		Meropenem	MEM	Oxoid
		Aztreonam	ATM	BioMaxima
		Cefoxitin	FOX	BioMaxima
	Other (glycopeptides, polymyxins)	Vancomycin	VA	Oxoid
		Teicoplanin	TEC	Oxoid
		Colistin sulfate	CT	Emapol
Inhibition of protein synthesis	Amino-glycosides	Gentamicin	CN	Oxoid
		Amikacin	AK	Oxoid
		Tobramycin	TOB	Oxoid
		Streptomycin	S	Oxoid
		Kanamycin	K	Oxoid
	Tetracyclines	Doxycycline	DO	Oxoid
		Tigecycline	TGC	Oxoid
		Tetracycline	TE	Oxoid
		Oxytetracycline	OT	Oxoid
	Oxazilidinones	Linezolid	LNZ	BioMaxima
	Streptogramins	Chinopristina/dalfopristin	SYN	BioMaxima
	Chloramphenicol		C	BioMaxima
	Macrolides	Erythromycin	E	Oxoid
	Lincosamides	Clindamycin	DA	BioMaxima
		Lincomycin	MY	Oxoid
	Fusidanes	Fusidic acid	FA	BioMaxima
DNA synthesis inhibitors	Fluoroquinolones	Norfloxacin	NOR	Oxoid
		Ciprofloxacin	CIP	Oxoid
		Marbofloxacin	FOR CYL	BioMaxima
		Flumequine	UB	Oxoid
Folic acid synthesis inhibitors	Sulfonamides with dihydrofolate reductase (DHFR) inhibitor	Trimethoprim-sulfamethoxazole	SXT	Oxoid
RNA synthesis inhibitors	Rifamycins	Rifampicin	RA	BioMaxima

## Data Availability

Not applicable.

## Supplementary Materials

The following are available online at https://www.mdpi.com/article/10.3390/antibiotics10111306/s1, Figure S1: Photographs visualizing plaque sizes and arrangement with the use of ampicillin in different variables of the DLA method, Figure S2: Photographs visualizing plaque sizes and arrangement with the use of cefotaxime in different variables of the DLA method.
