# Supplementary material for: Double-Layer Agar (DLA) Modifications for the First Step of the Phage-Antibiotic Synergy (PAS) Identification
Source: Antibiotics (Basel). 2021 Oct 26;10(11):1306. doi: 10.3390/antibiotics10111306 (PMC8614717; doi:10.3390/antibiotics10111306)
Supplement: Supplementary file 1 [file antibiotics-10-01306-s001.zip › antibiotics-1421921-supplementary.pdf]

Double-Layer Agar (DLA) Modifications for the First Step of the Phage-Antibiotic Synergy (PAS) Identification

Xymena Stachurska, Marta Roszak, Joanna Jabłońska, Małgorzata Mizielińska and Paweł Nawrotek

Supplementary Material

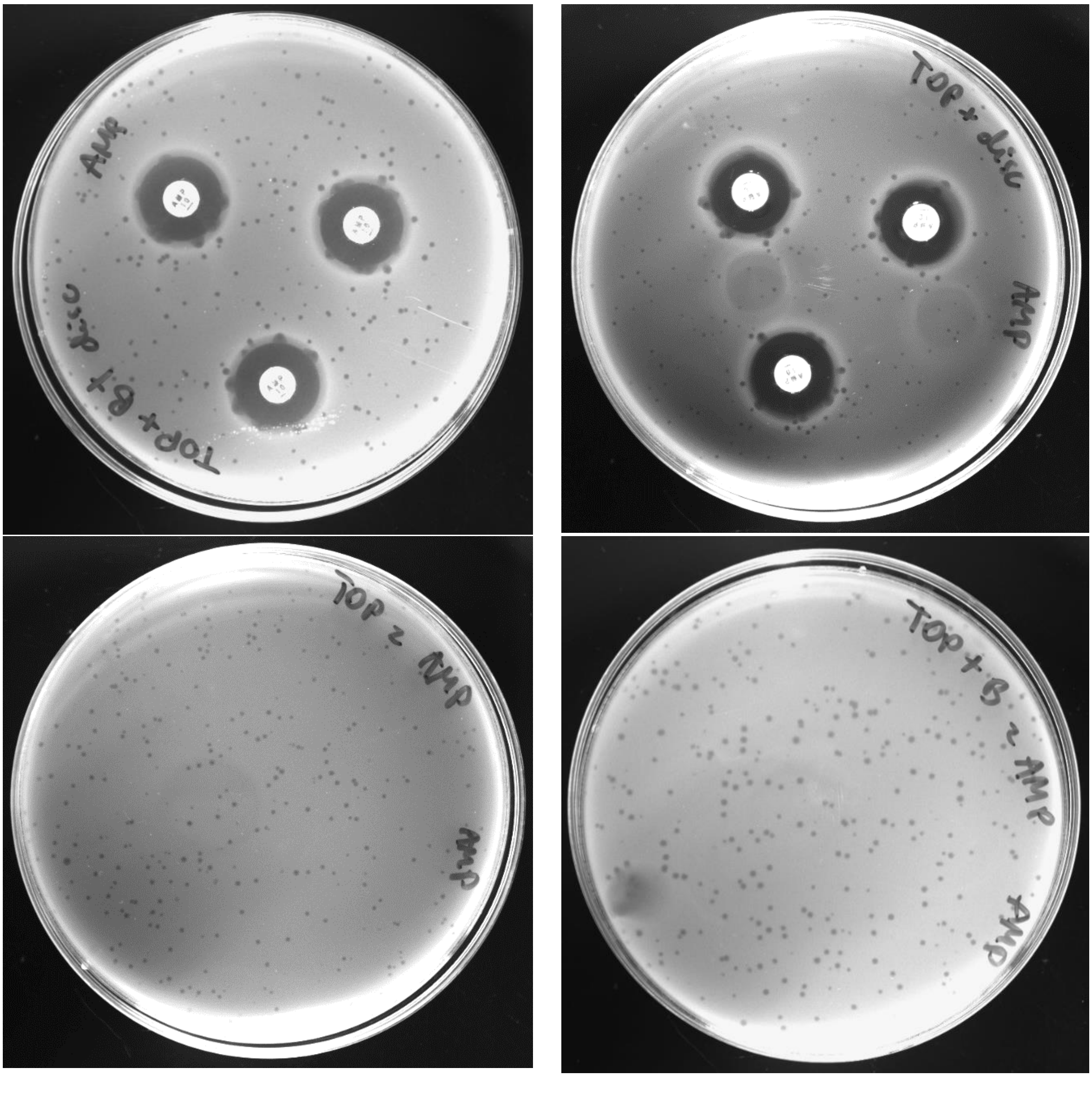

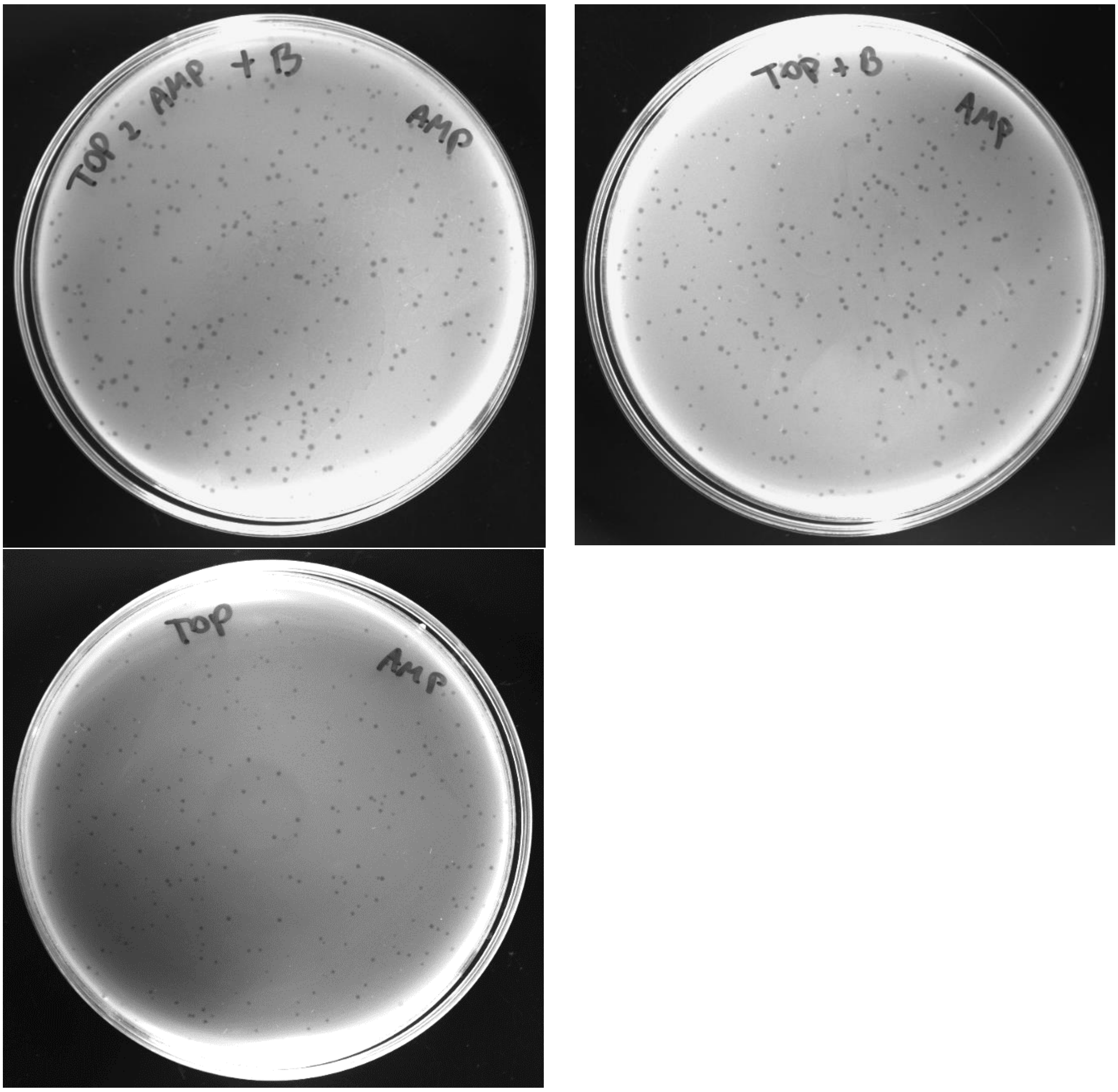

Figure S1. Photographs visualizing plaque sizes and arrangement with the use of ampicillin in different variables of the DLA method.

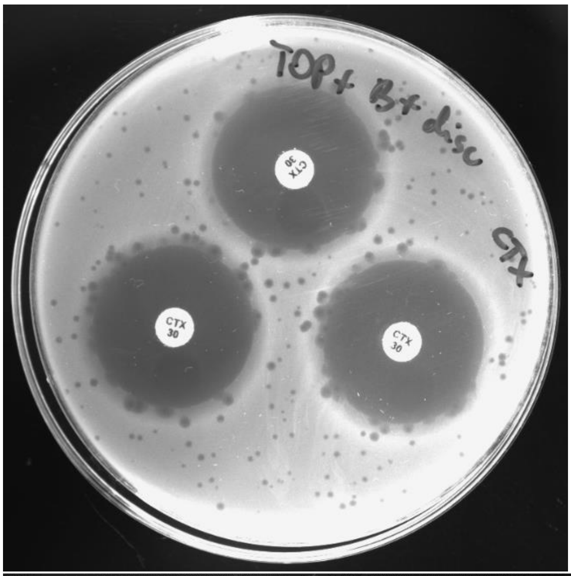
TOP + B + disc
CTX 30
CTX 30
CTX 30
CTX

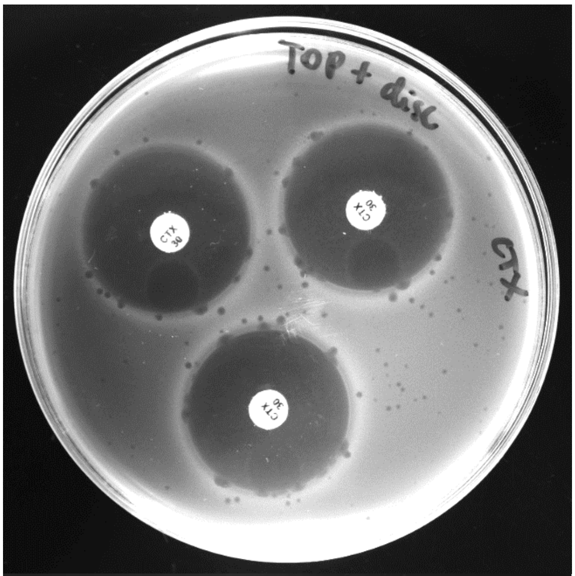
TOP + disc
CTX 30
CTX 30
CTX 30
CTX

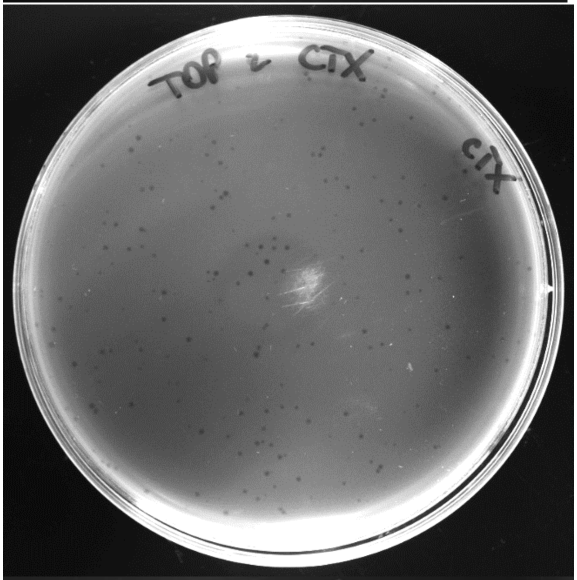
TOP 2 CTX
CTX

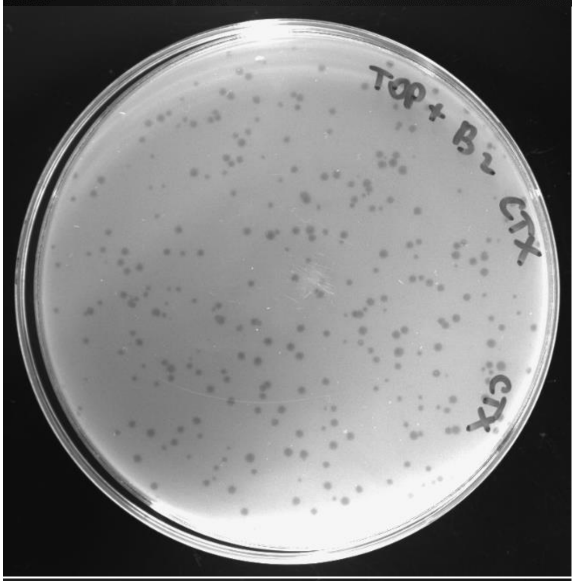
TOP + B 2 CTX
CTX

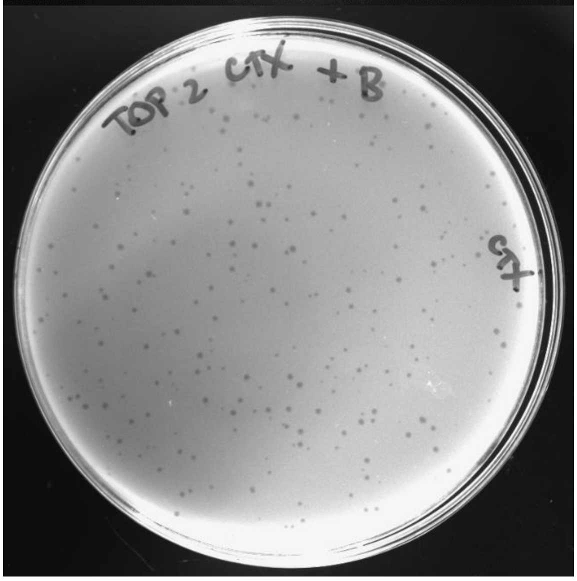
TOP 2 CTX + B
CTX

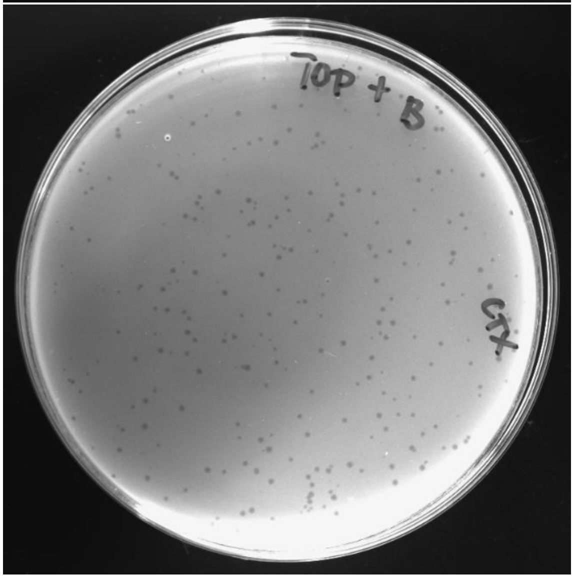
TOP + B
CTX

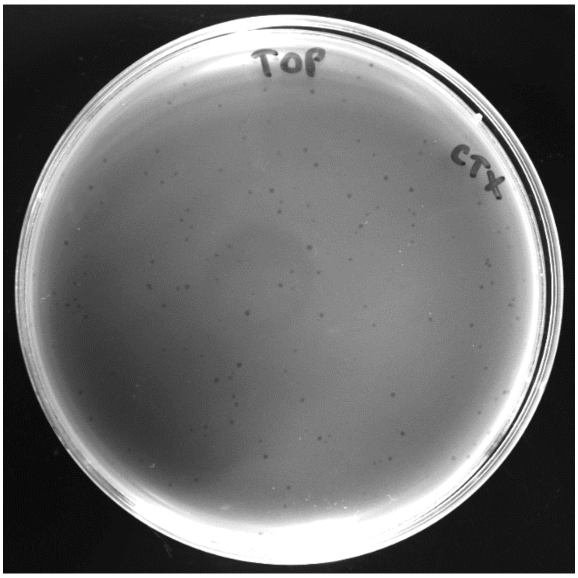

Figure S2 Photographs visualizing plaque sizes and arrangement with the use of cefotaxime in different variables of the DLA method.
